# Tuberculosis and Migration: A Challenge for Medical Staff and Public Health

**DOI:** 10.1155/2016/8186036

**Published:** 2016-11-14

**Authors:** Ines Griesshammer, David Shiva Srivastava, Christophe von Garnier, Till Silvan Blaser, Aris Exadaktylos, Moritz Steib

**Affiliations:** ^1^Emergency Department, University Hospital of Bern, Bern, Switzerland; ^2^Department of Pneumology, Tiefenau Spital Bern, Bern, Switzerland

## Abstract

A high number of asylum seekers enter Switzerland every year. They often originate from countries with a high TB prevalence. Our patient from Somalia presented with 2 lipoma-like tumors with pain on palpation on his left chest wall but no symptoms including coughing, fever, night-sweats, or loss of weight. CT scan then showed diffuse infiltrations of his lung and multiple abscesses on his left chest wall. Therefore contagious tuberculosis (TB) was suspected and the patient was put in isolation. In the follow-up the diagnosis of open TB was proofed with bronchial secretion and EBUS-guided biopsy that showed acid-fast rods. This particular case shows how difficult the identification of patients with open TB can be, especially if there are no respiratory or systemic symptoms. Therefore awareness of possible infectious disease is paramount for ED Doctors treating patients from countries with high prevalence. Early and strict isolation measures can help to reduce risk of contagion among staff and patients.

## 1. Introduction

According to the latest data of the Swiss Health Society (BAG; 08/2015), registered cases of TB in Swiss citizens are decreasing in the last years, whereas the numbers of infections in foreigners remain stable.

More cases of multiresistant forms, especially in asylum seekers and refugees, are found, making the treatment options more difficult [1].

In 2014 Switzerland had 23.800 cases of asylum requests [2] and it has been shown that this group of patients often originates from countries with a high TB prevalence [3].

## 2. Case Presentation

Our patient was sent by the temporary center for refugees to our ED. He had arrived in Switzerland 2 days earlier, travelling from Somalia via Libya and Italy. He spent 7 months in Libya on the streets under poor and unhygienic conditions and first noticed skin lesions then.

Approximately 2 months ago in Italy, he noticed a small node in his left axilla, for which he underwent needle aspiration. The results of this investigation are unfortunately unknown and our efforts to obtain the results remained futile for now.

In the last month he developed two more solid skin tumors which were painful on palpation.

In his surroundings there were no people with equal symptoms or people with known tuberculosis (TB). The patient denied other symptoms or complaints including coughing, shortness of breath, fever, night-sweats, or loss of weight.

We saw a hemodynamically stable and afebrile patient (BP 120/70 mmHg, HR 100/min, and T 36.2°C) in good condition. We did not find any lymphadenopathy but a wheezing sound on his left chest.

We found a hypochromic microcytic anemia (Hb 118 g/L) with thrombocytosis (497 G/L) and a normal leukocyte-count. CRP was only slightly elevated (CRP 15 mg/L).

On his chest wall there were 2 lipoma-like tumors (15 × 10 cm and 5 × 3 cm) with pain on palpation but no signs of inflammation.

The Ultrasound of the lesions showed an abscess-like configuration with fluid-filled chambers.

**Figure 1:**
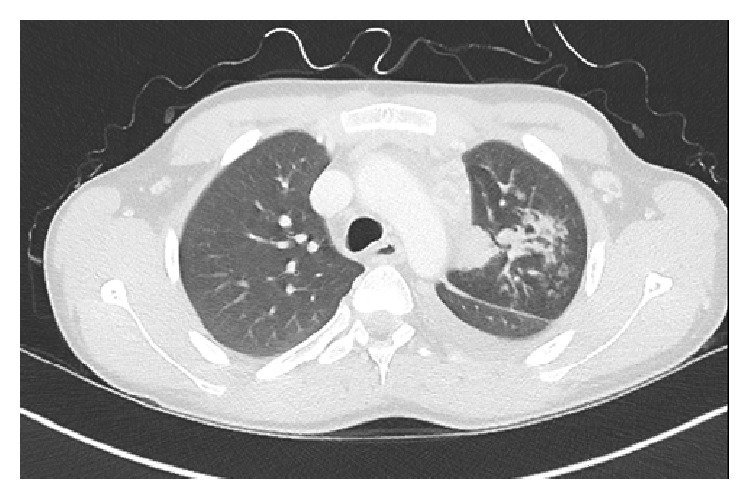
Diffuse infiltrations of the left upper lung/mediastinal abscesses.

**Figure 2:**
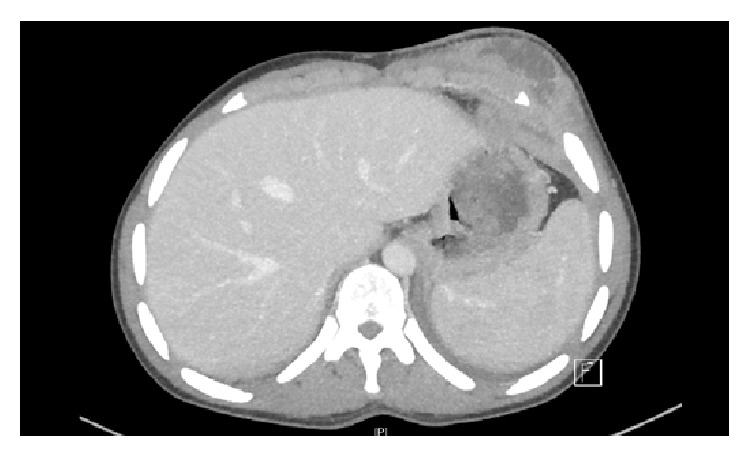
Multiple abscesses on the left chest wall with infiltrations of left ventral ribs.

**Figure 3:**
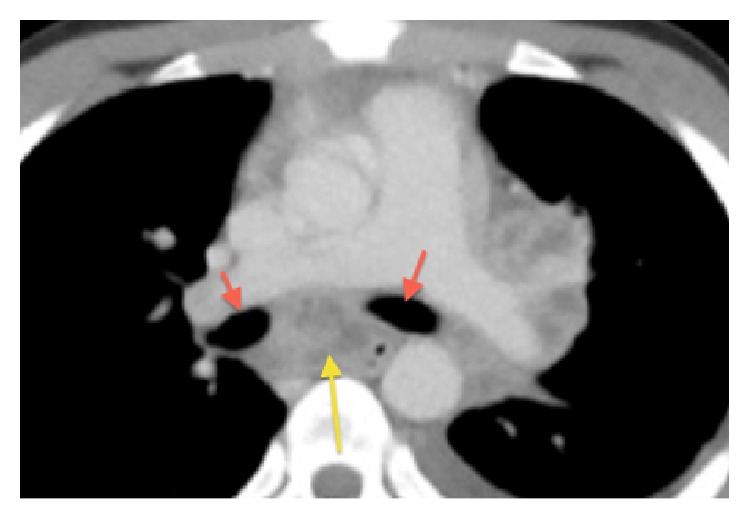
Compression of main bronchi by infracarinal lymph node/abscesses.

The CT scan of chest and abdomen revealed diffuse infiltrations of the left upper lung with pleural effusion, multiple left mediastinal abscesses (most likely originating from lymph-nodes), and multiple abscesses on the left chest wall with infiltrations of left ventral ribs 6–9 (Figures 1, 2, and 3).

As conclusion of our results we suspected a disseminated TB with pulmonary and subcutaneous lesions.

Open, contagious TB was not excluded and the patient was put under isolation and admitted to the pneumological ward. Serological tests for HIV and hepatitis remained negative.

**Figure 4:**
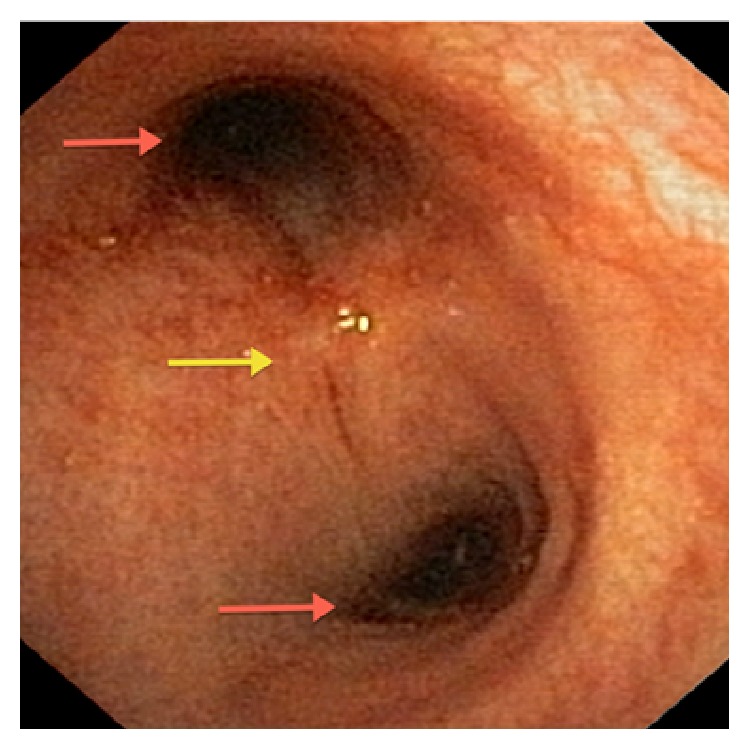
Bronchoscopy: broadened carina with compression of the main bronchi.

The patient underwent bronchoscopy (Figure 4). Bronchial secretion and EBUS-guided biopsy of the infracarinal lymph node/abscess showed acid-fast rods. In the biopsy additional TB typical granulomas could be seen. In the PCR no rifampicin-resistance-gene-mutation could be detected, and cultures are pending.

An antituberculotic therapy with rifampicin, isoniacide (additionally vitamin B6), pyrazinamide, and myambutol was started (planned duration of the therapy for 9 months because of the disseminated manifestations). To guarantee the intake the patient was transferred to a rehabilitation center with an expertise for tuberculosis treatment; afterwards a directly observed therapy (DOT) is performed in the refugee center.

## 3. Discussion

This case of a patient with subcutaneous TB but no other symptoms shows the broad spectrum of the new emergence of rare infectious diseases.

In our case open TB was found with direct proof of acid-fast rods and positive PCR in the bronchial secretion.

Identification of patients with active or open TB can often be difficult as only about one-third to one-half of the patients show the classical symptoms [4] with the rest being oligosymptomatic to asymptomatic [2].

The risk of infection is depending on various factors like concentration of mycobacteria in the air, the mycobacteria's virulence, the duration of exposure, and the individual predisposition.

Accordingly patients are contagious when having mycobacteria in their respiratory tract, if their secretion has a sufficient amount of mycobacteria and if it enters the air as an aerosol (typically in coughing patients) [5, 6].

The risk of contagion in noncoughing and only talking patients might be present if there is a high amount of mycobacteria involved but it is still extremely low [6].

This particular case shows how difficult the identification of patients with open TB is, especially if there are no respiratory symptoms or systemic symptoms like night-sweats, weight loss, or fever and even more if the communication with the patient is difficult due to language and cultural barriers.

Low threshold and raised awareness for possible infectious disease are paramount for ED Doctors treating patients from countries with high prevalence. Early and strict isolation measures can help to reduce risk of contagion among staff and patients. If diagnosed, it is a further challenge in patients to secure the intake of their antituberculotic medication to avoid appearance of mycobacterial resistance and the risk of further infections.
